# TARP γ-2 Is Required for Inflammation-Associated AMPA Receptor Plasticity within Lamina II of the Spinal Cord Dorsal Horn

**DOI:** 10.1523/JNEUROSCI.0772-16.2017

**Published:** 2017-06-21

**Authors:** Steve J. Sullivan, Mark Farrant, Stuart G. Cull-Candy

**Affiliations:** Department of Neuroscience, Physiology and Pharmacology, University College London, London WC1E 6BT, United Kingdom

**Keywords:** AMPARs, dorsal horn, inflammation, spinal cord, stargazin, TARPs

## Abstract

In the brain, transmembrane AMPAR regulatory proteins (TARPs) critically influence the distribution, gating, and pharmacology of AMPARs, but the contribution of these auxiliary subunits to AMPAR-mediated signaling in the spinal cord remains unclear. We found that the Type I TARP γ-2 (stargazin) is present in lamina II of the superficial dorsal horn, an area involved in nociception. Consistent with the notion that γ-2 is associated with surface AMPARs, CNQX, a partial agonist at AMPARs associated with Type I TARPs, evoked whole-cell currents in lamina II neurons, but such currents were severely attenuated in γ-2-lacking *stargazer* (*stg/stg*) mice. Examination of EPSCs revealed the targeting of γ-2 to be synapse-specific; the amplitude of spontaneously occurring miniature EPSCs (mEPSCs) was reduced in neurons from *stg/stg* mice, but the amplitude of capsaicin-induced mEPSCs from C-fiber synapses was unaltered. This suggests that γ-2 is associated with AMPARs at synapses in lamina II but excluded from those at C-fiber inputs, a view supported by our immunohistochemical colabeling data. Following induction of peripheral inflammation, a model of hyperalgesia, there was a switch in the current-voltage relationships of capsaicin-induced mEPSCs, from linear to inwardly rectifying, indicating an increased prevalence of calcium-permeable (CP) AMPARs. This effect was abolished in *stg/stg* mice. Our results establish that, although γ-2 is not typically associated with calcium-impermeable AMPARs at C-fiber synapses, it is required for the translocation of CP-AMPARs to these synapses following peripheral inflammation.

**SIGNIFICANCE STATEMENT** In the brain, transmembrane AMPAR regulatory proteins (TARPs) critically determine the functional properties of AMPARs, but the contribution of these auxiliary subunits to AMPAR-mediated signaling in the spinal cord remains unclear. An increase in the excitability of neurons within the superficial dorsal horn (SDH) of the spinal cord is thought to underlie heighted pain sensitivity. One mechanism considered to contribute to such long-lived changes is the remodeling of the ionotropic AMPA-type glutamate receptors that underlie fast excitatory synaptic transmission in the SDH. Here we show that the TARP γ-2 (stargazin) is present in SDH neurons and is necessary in a form of inflammatory pain-induced plasticity, which involves an increase in the prevalence of synaptic calcium-permeable AMPARs.

## Introduction

AMPA-type glutamate receptors (AMPARs) mediate fast excitatory synaptic transmission in the superficial dorsal horn (SDH) of the spinal cord (lamina I and II). Lamina II contains a heterogeneous population of excitatory and inhibitory interneurons that receive glutamatergic input from peripheral pain fibers and from local interneurons (Todd, 2010). These lamina II cells shape the excitatory output of lamina I neurons that project to the brain, making them critical in the processing of noxious stimuli (Wang et al., 2013; Yasaka et al., 2014). Following peripheral injury or inflammation, SDH neurons exhibit changes in surface AMPAR expression (Park et al., 2009; Kopach et al., 2011) that are thought to contribute to central sensitization and heightened pain sensitivity (Latremoliere and Woolf, 2009).

AMPARs are tetrameric assemblies formed from the subunits GluA1–4 (Traynelis et al., 2010). In the SDH, the most abundant AMPAR subunits are GluA1 and GluA2 (Hartmann et al., 2004; Nagy et al., 2004; Polgár et al., 2008). Although the existence of GluA2-lacking, calcium-permeable (CP), AMPARs has been observed in specific SDH neurons of young animals (Nagy et al., 1994; Engelman et al., 1999; Tong and MacDermott, 2006), many of the AMPARs in mature SDH neurons contain GluA2 subunits (Nagy et al., 2004; Polgár et al., 2008), rendering them calcium impermeable (CI-AMPARs) (Katano et al., 2008). By contrast, following peripheral injury or inflammation, CP-AMPARs are upregulated at synaptic (Katano et al., 2008; Vikman et al., 2008; Park et al., 2009; Chen et al., 2013) and potentially extrasynaptic (Kopach et al., 2011) sites in SDH neurons. Such plasticity may occur via NMDAR-dependent activation of protein kinase C and subsequent phosphorylation of GluA2 (Park et al., 2009) and GluA1 (Wang et al., 2011). Indeed, block of CP-AMPARs (Sorkin et al., 1999) or the genetic deletion of GluA1 attenuates the development of hyperalgesia, whereas deletion of GluA2 has the opposite effect (Hartmann et al., 2004), suggesting that CP-AMPAR expression is important in pain-related plasticity.

The functional properties of AMPARs depend not only on their subunit composition but also on the presence of associated auxiliary proteins. Of these, the transmembrane AMPAR regulatory proteins, classified as Type I (γ-2, γ-3, γ-4, and γ-8) or Type II (γ-5 and γ-7) transmembrane AMPAR regulatory proteins (TARPs), have received particular attention (Jackson and Nicoll, 2011b). TARPs influence the trafficking, pharmacology, channel gating, and conductance of AMPARs (Coombs and Cull-Candy, 2009; Jackson and Nicoll, 2011b) and are implicated in synaptic plasticity (Rouach et al., 2005; Tomita et al., 2005a; Jackson and Nicoll, 2011a).

In the SDH of the spinal cord, immunolabeling has revealed the presence of multiple TARP isoforms (Larsson et al., 2013). Although the precise role of different TARPs in excitatory transmission within the SDH has not been explored, knockdown of γ-2 has been shown to lessen the severity of hyperalgesia (Tao et al., 2006), possibly by preventing the increased surface expression of CP-AMPARs (Guo et al., 2014). Moreover, polymorphisms in the γ-2 gene *CACNG2* have been associated with susceptibility to chronic pain in humans (Nissenbaum et al., 2010).

Using pharmacological approaches, immunohistochemistry and patch-clamp recording from SDH neurons in acute spinal slices from wild-type (wt) and γ-2-lacking *stargazer* (*stg/stg*) mice, we show that most lamina II neurons express γ-2-associated AMPARs. In these cells, EPSCs arising from local inputs, but not those arising from peripheral pain fibers activated by capsaicin, were attenuated in *stg/stg* mice. In addition, we found that the presence of γ-2 is necessary for CP-AMPAR plasticity in a model of inflammatory hyperalgesia. Our studies establish an important role for γ-2 in shaping dorsal horn excitability in both normal and pathological pain states.

## Materials and Methods

### 

#### 

##### Animals.

Experiments were performed using tissue from male and female wt (C57BL/6), *stargazer* (*stg/stg*), and GAD65-eGFP mice, postnatal days 21 and 30 (P21-P30). Both *stg/stg* and GAD65-eGFP mice were on a C57BL/6 background. The former were bred from +/*stg* mice and identified based on their characteristic phenotype (head tossing and ataxic gate). Phenotypic identification was confirmed by genotyping (Letts et al., 1998). GAD65-eGFP mice (López-Bendito et al., 2004) were maintained as homozygotes. All procedures for the care and treatment of mice were in accordance with the Animals (Scientific Procedures) Act 1986.

##### Western blots.

CNS tissue was rapidly dissected in ice-cold PBS and placed in RIPA lysis buffer (Thermo Scientific) with protease inhibitor (Roche) and 1% IgPal detergent (Sigma). Volumes of lysis buffer were adjusted for the source of tissue (spinal cord 250 μl; cerebellum or cortex 1000 μl; hippocampus 250 μl). Tissue was lysed by applying ∼20–30 strokes with a Dounce homogenizer. Following gentle rotation at 4°C for 1 h, samples were ultracentrifuged at 35,000 rpm for 35 min at 4°C. Pellets were discarded, and the protein content of the supernatant was measured using a Bradford assay (Bio-Rad). Samples were diluted to equal concentrations (8 mg/ml) and reacted with equal volumes of 2× Laemmli buffer (Sigma) at 65°C for 15 min. A total of 80 μg of protein was separated in a 30% polyacrylamide Tris glycine gel and transferred onto nitrocellulose paper. Blots were blocked for 1 h in PBS containing 4% milk and 0.05% Tween and reacted with primary antibodies overnight at 4°C. Following several washes, blots were then incubated with 1:1000 HRP-conjugated secondary antibody in blocking solution for 1 h. Blots were washed and protein bands were detected using a chemiluminescent assay (Pierce Thermo Scientific), imaged with a digital camera (Bio-Rad). Protein sizes were interpolated from a standard ladder (Sigma).

##### Immunohistochemistry.

Mice were deeply anesthetized with an intraperitoneal injection of ketamine (100 mg/kg)/xylazine (10 mg/kg) and perfused transcardially with ice-cold PBS followed by 4% (w/v) formaldehyde in PBS. Brains and spinal cords were removed and incubated overnight at 4°C in 4% formaldehyde. The following day, tissues were exposed to a series of increasing sucrose concentrations (10%, 20%, and 30% w/v). Tissue was embedded in a cutting compound consisting of 11% polyvinyl alcohol and 5% carbowax and frozen in dry ice before sectioning. Cryosections (20 μm) were adhered to charged glass slides and stored at −20°C. All spinal cord sections were transverse, from L3 to L6. Cerebellar sections were sagittal, and hippocampal sections were coronal. Before immunolabeling, slides were washed in PBS.

Antigen retrieval was tested for all antibodies under varying durations of pepsin exposure, as described previously (Watanabe et al., 1998; Nagy et al., 2004). Briefly, slides were incubated in 0.4% (w/v) pepsin (Dako) in 0.2 m HCl at 37°C. Seven to 10 min of pepsin treatment proved optimal for AMPARs and γ-2, whereas γ-7 and γ-8 antibodies appeared to label best without pepsin treatment (Table 1). Samples were blocked and permeabilized for 1 h in PBS containing the following: 10% serum, 0.5% Triton, and 0.5% BSA. The source of the serum was matched to the species in which the secondary antibodies were raised. Slides were incubated in blocking solution containing primary antibody overnight at 4°C. The next day, sections were washed and treated with fluorescent secondary antibodies in blocking solution. After washing, sections were treated with DAPI (Thermo Scientific, 1:1000) for 10 min, dried, and mounted in ProLong Gold antifade (Thermo Scientific). To label lamina II, biotin-linked IB4 (Thermo Scientific) (10 μg/ml) was coincubated with the primary antibodies, whereas Avidin 555 (2 μg/ml) (Thermo Scientific) was coincubated with secondary antibodies. The following day sections were imaged using a Leica TCS SPE confocal microscope.

**Table 1.** Details of the primary antibodies used in this study and the requirement for antigen retrieval (pepsin treatment)*^a^*

Antigen	Species	Source	Number	Dilution (Western)	Dilution (IHC)	Pepsin (IHC)
γ-2	Guinea pig	M. Watanabe	—	1:500	—	—
γ-2*^b^*	Rabbit	Cell Signaling Technology	8511	—	1:200	Yes*^c^*
γ-3	Guinea pig	M. Watanabe	—	1:500	—	—
γ-4	Rabbit	Abcam	ab81107	1:500	—	—
γ-5	Rabbit	M. Watanabe	—	1:500	—	—
γ-7	Rabbit	Frontiers Institute	Af720	1:500	1:500	No
γ-8	Rabbit	Frontiers Institute	Af1000	1:2000	1:1000	No
GluA1	Rabbit	Abcam	ab31232	—	1:500	Yes
GluA2	Mouse	Millipore	MAB397	1:500	1:500	Yes
c-Fos	Goat	Santa Cruz Biotechnology	sc-52-G	—	1:100	No
Actin	Mouse	Abcam	ab3280	1:5000	—	—
GFP	Chicken	Abcam	Ab13970	—	1:500	No
CGRP*^b^*	Mouse	Sigma	C7113	—	1:400	No
vGluT1*^b^*	Guinea pig	Synaptic Systems	135 304	—	1:400	No
vGluT2*^b^*	Guinea pig	Millipore	AB2251	—	1:4000	No

*^a^*IHC, Immunohistochemistry.

*^b^*Used for imaging synapses (see Materials and Methods).

*^c^*Pepsin used for γ-2 labeling of PFA fixed tissue but not with carbodiimide fix (synaptic imaging).

##### Antibodies.

Details regarding the primary antibodies are provided in Table 1. We tested each TARP antibody by running Western blots on lysates from HEK cells expressing each TARP isoform independently. For Western blots, we used HRP-conjugated secondary antibodies raised in goat: anti-mouse (Santa Cruz Biotechnology 2005, 1:1000), anti-guinea pig (Santa Cruz Biotechnology 2438, 1:1000), and anti-rabbit (Rockland 611–1302, 1:4000). For immunohistochemistry, we used secondary antibodies conjugated to Alexa dyes (488, 555, and 647) raised against rabbit, mouse, guinea pig, chicken, and goat (for example, see Thermo Scientific, A-11008). All anti-rabbit, -mouse, -guinea pig, and -chicken fluorescent antibodies were raised in goat whereas anti-goat antibodies were raised in donkey.

##### Preparation of spinal cord slices for electrophysiological recording.

Spinal cords were rapidly dissected and sectioned in ice-cold slicing solution, which contained the following (in mm): 120 K-gluconate, 15 KCl, 0.05 EGTA, 20 HEPES, 4 pyruvate, 25 glucose, 10 Na ascorbate, 0.05 d-AP5 (pH 7.4, bubbled with 100% O_2_). Sections (400 μm) were obtained from L3-L6 spinal cord using a Campden vibrotome (7000 smz) with a ceramic blade. Slices were then heated in recovery solution (35°C; bubbled with 95% O_2_, 5% CO_2_) for 30–60 min before recording; this solution was similar to that used for whole-cell recordings (see below), except it contained high Mg^2+^ (6 mm) and low Ca^2+^ (0.5 mm).

##### Whole-cell recordings.

All recordings were performed at room temperature (22°C-25°C). Slices were transferred to a submerged recording chamber and perfused with a solution containing the following (in mm): 125 NaCl, 2.5 KCl, 26 NaHCO_3_, 1.25 NaH_2_PO_4_, 25 glucose, 2 CaCl_2_, and 1 MgCl_2_. Slices were viewed using a Zeiss Axioscop with a 4× objective, and the substantia gelatinosa of the horn identified. Under 40× magnification with differential interference contrast imaging, lamina II was identified as a translucent band between the relatively darker laminae (I and III). Cells within the outer half of lamina II with soma diameters between 9 and 12 μm were targeted for recording.

For voltage-clamp recordings, the intracellular solution contained the following (in mm): 128 CsCl, 2 NaCl, 1 CaCl_2_, 10 HEPES, 10 EGTA, 10 TEA-Cl, 2 MgATP, and 1 *N*-(2,6-dimethylphenylcarbamoylmethyl) triethylammonium bromide (QX314; Tocris Bioscience) (adjusted to pH 7.4 with CsOH). In experiments where rectification was measured, 100 μm spermine tetrahydrochloride (Sigma) was included in the pipette solution. For current-clamp recordings, the intracellular solution contained the following (in mm): 120 K-gluconate, 10 KCl, 40 HEPES, 0.5 EGTA, 3 MgCl_2_, 2 NaATP, and 0.3 NaGTP (adjusted to pH 7.4 with KOH). Series resistance, input capacitance, and input resistance were determined by measuring the current elicited by a 5 mV hyperpolarizing voltage step. The series resistance remaining after 60%–80% compensation was typically <8 mΩ. Recordings were made with an Axopatch 200A amplifier, low-pass filtered (Bessel 10 kHz), and sampled at 20 kHz using a Digidata 1440A with pClamp10 software (Molecular Devices).

AMPAR-mediated currents were pharmacologically isolated by the addition of 10 μm 2-(3-carboxypropyl)-3-amino-6-(4 methoxyphenyl)pyridazinium bromide (SR-95531; gabazine), 10 μm strychnine, and 50 μm d-AP5. To activate C-fibers, slices were exposed to 0.1 μm capsaicin (diluted from 50 mm stock in DMSO). TTX (1 μm) was included in the recording solution when recording miniature EPSCs (mEPSCs), or the responses to bath applied agonists [25 μm AMPA or 10 μm CNQX, both with 100 μm cyclothiazide]. Quantal EPSCs (qEPSCs) were evoked in the presence of Sr^2+^ (replacement of CaCl_2_ with 5 mm SrCl_2_) using a thin-walled borosilicate glass micropipette filled with extracellular solution (0.5–1 mΩ) placed in the dorsal horn ∼50 μm away from the recorded cell. The minimal voltage and frequency necessary to produce reliable responses were used (typically 5–30 V, 0.1 ms, 0.05–0.2 Hz).

##### Recordings from GABAergic interneurons.

Lamina II GAD65-eGFP-positive cells were identified under epifluorescence using LED illumination (pE-100; CoolLED). To confirm the identity of each recorded GAD-positive cell, we included biocytin (0.3% w/v, Thermo Scientific) in the pipette solution. At the end of the recording, the holding potential was switched to 0 mV and the pipette was slowly retracted. Slices were immediately placed in 4% PFA in PBS and left overnight at 4°C. Following three 10 min washes in PBS, slices were incubated in Triton (0.1%) and Avidin 555 (2 μg/ml) for 4 h at 4°C. Slices were then washed, reacted with DAPI (5 μg/ml), dried, and mounted in ProLong Gold antifade overnight. The following day, confocal images of cells were taken with a 63× objective. GFP expression in the filled neurons was confirmed by detection of fluorescence and/or GFP immunoreactivity.

##### Synaptic immunolabeling and analysis.

When using our transcardial fixation protocol (see above), the extent of pepsin treatment required to reveal γ-2 labeling often proved too harsh to preserve the labeling of presynaptic markers. Therefore, to reduce the fixation time and improve antigen retrieval, we fixed brain slices prepared as in our whole-cell recording experiments. In some cases, slices were incubated in 4% PFA in PBS (1 h), resectioned using a cryostat, and treated with pepsin (1 min), as described above. In most cases, however, we adopted a light fixation protocol using carbodiimide (Moffett et al., 1993) rather than PFA, allowing us to avoid the use of pepsin altogether. Slices were fixed for 1.5 h in a solution containing 6% carbodiimide, 1 mm *n*-hydroxyl succinimide, and 200 mm sucrose. All blocking solutions, antibody solutions, and mounting media were identical to those used with PFA. Following fixation, slices were washed and incubated in blocking/permeablization solution for 2 h. Slices were treated with primary antibodies for 48 h, washed, and then treated with secondary antibodies for 24 h. Confocal images (1024 × 1024 pixels; 56.2 × 56.2 μm) of synaptic puncta were acquired with a 63×, 1.3 numerical aperture, oil-immersion objective under 3× zoom. For analysis, *Z*-stacks consisting of six images (each 252 nm apart) were deconvolved using ImageJ plugins developed by the Biological Imaging Group (http://bigwww.epfl.ch/). Theoretical point spread functions were generated using the Born and Wolf algorithm (PSF Generator plugin) and deconvolution performed using the Landweber algorithm (Deconvolutionlab plugin). A maximum intensity projection of the deconvolved images with contrast enhancement was used to detect synaptic puncta. Data from pepsin and carbodiimide treated tissue were pooled.

The proportion of postsynaptic puncta contacting or juxtaposed to any number presynaptic puncta (and vice versa) was determined using CellProfiler software (version 2.1.1; http://cellprofiler.org/) (Carpenter et al., 2006; Kamentsky et al., 2011). We used the adaptive thresholding object detection algorithm to identify puncta separately on each imaging channel. To detect overlapping or adjacent presynaptic or postsynaptic puncta, object edges were uniformly expanded by one pixel and overlaid with the objects of the opposing channel for comparison. An object contacting any number of objects on the opposing channel was considered positive for “overlap”. A control image, rotated 180 degrees, was used to estimate the chance likelihood of object overlap (false positive rate). This background value was subtracted to obtain the final values reported in the text.

##### Induction of peripheral inflammation.

Peripheral inflammation was induced by complete Freund's adjuvant (CFA), an oil emulsion containing 1 mg/ml *Mycobacterium tuberculosis* (Sigma). Mice received a unilateral subcutaneous intraplantar injection of 20 μl CFA. For histological detection of c-Fos, mice were perfused transcardially with 4% PFA 1.5 h after CFA injection, when c-Fos expression was highest. Electrophysiological recordings were performed 24 h after CFA injection. At the time of slice preparation (see below), the side of the slice ipsilateral to the injection was marked with a small notch in the white matter of the ventral horn for identification during recordings.

##### Analysis of electrophysiological data.

All EPSC analysis was performed using Igor Pro (version 6.34a, Wavemetrics) with Neuromatic (version 2.8v, http://www.neuromatic.thinkrandom.com/). For EPSC detection, records were digitally filtered offline at 1 kHz and a threshold crossing procedure used (Kudoh and Taguchi, 2002). The thresholds were typically 8 pA at −60 or 60 mV or 6 pA at 40 mV. For each cell, the average EPSC waveform was constructed from all events (aligned at their onset) and its rise time (RT_20%–80%_) and decay (single exponential τ_deacy_) measured. The rectification of EPSCs was assessed by dividing the peak conductance of the average event at positive potentials (40 or 60 mV) by the conductance at −60 mV, assuming a reversal potential of 0 mV (RI_40/−60_ or RI_60/−60_). The rectification of whole-cell currents evoked by bath application of AMPA was calculated as the ratio of the slope conductances (RI_slope_; 35 to 40 mV/−55 to −60 mV). As the tonic whole-cell currents induced by bath application of CNQX were sometimes small, the holding current was measured using an automated procedure written in Igor Pro, as described previously (Rigby et al., 2015). Briefly, an all-point histogram was constructed from 1 s segments of the complete record (∼15 min). For each histogram, a single-sided Gaussian was fitted to the most-positive current values to remove the influence of phasic synaptic events, and the peak of the Gaussian was taken as the mean current value.

##### Peak-scaled nonstationary fluctuation analysis (psNSFA).

The weighted-mean single-channel conductance of synaptic receptors underlying qEPSCs and mEPSCs was measured using psNSFA (Traynelis et al., 1993). The peak of the average event was scaled to the peak of each individual event and subtracted. The residual noise was spilt into 30 bins of equal amplitude, and the average variance was computed within each bin and plotted against the corresponding average amplitude. The parabolic relationship between variance and amplitude was fitted using the following equation:

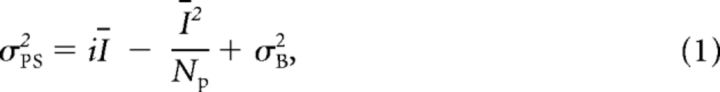
 where σ_PS_^2^ is the peak-scaled variance, *Ī* is the mean current, *i* is the weighted mean single-channel current, *N*_P_ is the number of channels open at the peak of the EPSC, and σ_B_^2^ is the background variance.

##### Statistics and data presentation.

Summary data are presented in the text as mean ± SEM. from *n* cells. Analyses of single datasets were conducted using a two-sided one-sample *t* test, with a null hypothesis that the mean equals zero. Comparisons involving only two datasets were performed using a two-sided two-sample Welch *t* test that does not assume equal variance. Analyses involving data from three or more groups were performed using either two-way ANOVA (Welch heteroscedastic *F* test), followed by pairwise comparisons using two-sided Welch two-sample *t* tests with Holm's sequential Bonferroni correction, or Kruskal-Wallis one-way ANOVA, followed by pairwise Wilcoxon rank sum tests with Holm's sequential Bonferroni correction. Paired data were compared with a two-way “split plot” ANOVA followed by two-sided Welch two-sample paired *t* tests. Exact *p* values are presented to two significant figures, except when *p* < 0.0001. Differences were considered significant at *p* < 0.05. Statistical tests were performed using R (version 3.3.2; R Foundation for Statistical Computing; http://www.r-project.org/) and R Studio (version 1.0.136; RStudio). The results of omnibus tests are given in the text or figure legends, with pairwise *p* values reported in the text and designated by asterisks in the figures.

## Results

### TARP expression in the spinal cord

**Figure 1.**
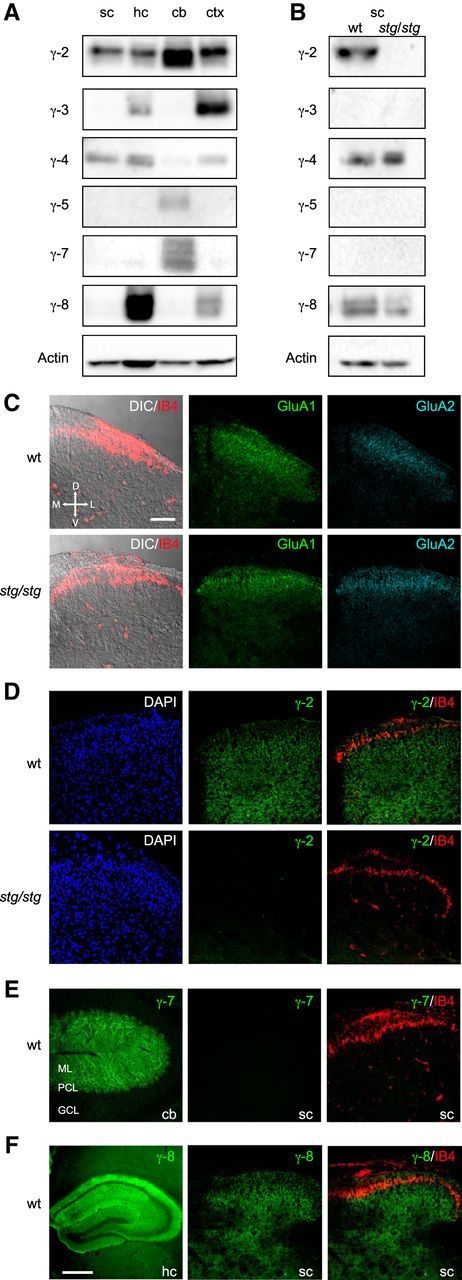
TARP expression in the spinal cord. ***A***, Immunoblots comparing tissue expression of γ-2, γ-3, γ-4, γ-5, γ-7, and γ-8 in homogenates of spinal cord (sc), hippocampus (hc), cerebellum (cb), and cortex (ctx). Note the presence of γ-2 and γ-4 in spinal cord. ***B***, Immunoblots comparing wt and *stg/stg* spinal cord. Note the expected absence of γ-2. Increased exposure times (compared with ***A***) were used for γ-3, γ-5, γ-7, and γ-8 and revealed the presence of γ-8 in both wt and *stg/stg* samples. The approximate molecular weights of the various TARPs (in kDa) were as follows: γ-2, 35; γ-3, 32; γ-4, 45; γ-5, 27; γ-7, 28; γ-8, 47. Actin (41 kDa) served as a loading control. ***C–F***, Confocal images (maximum intensity projections) of representative tissue sections immunolabeled for TARPs and AMPARs. ***C***, Dorsal horn of the spinal cord. Left, differential interference contrast images overlaid with fluorescence signal for the lamina II-specific marker IB4 (red). Middle and right panels, Similar pattern of expression for AMPAR subunits GluA1 (green) and GluA2 (cyan) in sections from wt and *stg/stg* mice. Here, and in following panels, all spinal cord images are transverse and shown in the same orientation. D, Dorsal; V, ventral; M, medial; L, lateral. Scale bar, 100 μm. ***D***, Comparison of γ-2 expression in the dorsal horn of wt and *stg/stg* mice. Left, DAPI staining (blue). Middle, γ-2 (green). Right, Overlay of IB4 (red) and γ-2 (green). ***E***, Left, γ-7 expression (green) in cerebellum (cb); molecular layer (ML), Purkinje cell layer (PCL), and granule cell layer (GCL). Middle, γ-7 (green) in spinal cord. Right, Overlay of IB4 (red) and γ-7 (green). ***F***, Left, γ-8 (green) in hippocampus (hc). Scale bar, 500 μm. Middle, γ-8 (green) in dorsal horn. Right, Overlay of IB4 (red) and γ-8 (green).

Using Western blots, we initially examined the expression of the complete family of TARPs (γ-2, γ-3, γ-4, γ-5, γ-7, and γ-8) in spinal cord homogenates from wt and *stg/stg* mice. To validate our antibodies, we also used tissue from three other brain regions (hippocampus, cerebellum, and cortex) for which TARP expression has been well characterized (Fig. 1*A*). We detected protein bands for γ-2, γ-4, and γ-8 in spinal cord but found no evidence for γ-3, γ-5, or γ-7. As expected, γ-2 was also present in all other brain regions examined, with the strongest expression observed in cerebellum (Tomita et al., 2003). The specificity of the γ-2 antibody was confirmed by the absence of signal in spinal cord samples from *stg/stg* mice (Fig. 1*B*). We found γ-4 in spinal cord as well as cortex and hippocampus, with a lower level in cerebellum (Tomita et al., 2003). We initially detected little or no γ-8 in spinal cord or cerebellum, despite a strong signal in hippocampus and a slightly weaker signal in cortex, as reported previously (Tomita et al., 2003). However, in separate immunoblot experiments where we examined spinal cord samples following a longer exposure, γ-8 was clearly present in tissue from both wt and *stg/stg* mice (Fig. 1*B*). Failure to detect γ-3, γ-5, and γ-7 in spinal cord did not reflect failure of the antibodies, as γ-3 was detected in cortex and, to a lesser extent, in hippocampus. Moreover, both γ-5 and γ-7 were detected in cerebellum, as previously described (Fukaya et al., 2005; Soto et al., 2009; Yamazaki et al., 2010). Of note, aside from the loss of γ-2, we detected no obvious change in the expression of other TARPs in spinal cord samples from *stg/stg* mice.

We next examined the spatial distribution of TARPs and AMPARs within the dorsal horn by performing immunohistochemistry on transverse spinal cord sections (Fig. 1*C–F*). To identify lamina II, we used Isolectin B4 (IB4), which labels a thin band of nonpeptidergic C-fibers in this lamina (Silverman and Kruger, 1990). Following antigen retrieval with pepsin (see Materials and Methods; Table 1), we were able to detect immunoreactivity for the AMPAR subunits GluA1 and GluA2, which showed relatively intense expression in the in spinal cord SDH. No obvious abnormalities were apparent in the laminar patterning of these AMPAR subunits in sections from *stg/stg* mice (Fig. 1*C*). Within the SDH and deeper laminae of wt mice, we detected punctate immunoreactivity for γ-2 (Fig. 1*D*) (Tao et al., 2006) but no immunoreactivity for γ-7 (Fig. 1*E*). However, we found pronounced γ-7 expression in sagittal sections of cerebellum, with the strongest signal in Purkinje cell bodies and the molecular layer, confirming the effectiveness of our protocol (Yamazaki et al., 2010). As expected, immunolabeling for γ-8 was readily detected in the hippocampus (Fig. 1*F*). In the spinal cord, immunoreactivity for γ-8 appeared punctate in lamina I and relatively diffuse in outer lamina II (Fig. 1*F*), as previously described (Larsson et al., 2013). We were unable to conclusively determine the expression pattern of γ-3, γ-4, or γ-5. The whole-serum γ-4 antibody used in Western blots appeared to label nonspecifically in spinal cord sections. It also failed to label brain sections, as did our antibodies for γ-3 and γ-5 (data not shown). Nonetheless, both our immunohistochemical and Western blot data indicate the clear presence of γ-2 in the SDH, and a lesser but clear presence of γ-8.

### TARP γ-2 is associated with functional AMPARs in lamina II neurons

**Figure 2.**
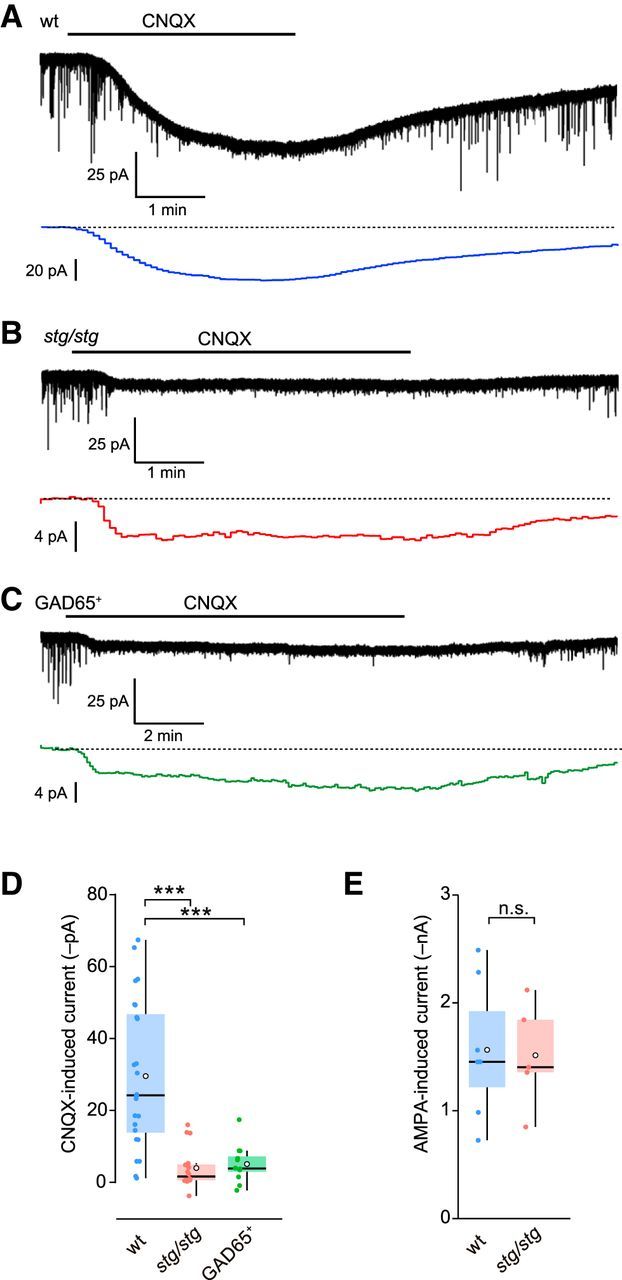
CNQX-evoked currents are reduced in lamina II neurons of *stg/stg* mice. ***A***, Representative whole-cell current (−60 mV) in a wt lamina II neuron evoked by bath application of CNQX (10 μm) in the presence of cylothiazide (100 μm). Bottom trace (colored), Isolated holding current (1 s bins) (see Materials and Methods). ***B***, Same as ***A***, but for a neuron from an *stg*/*stg* mouse. ***C***, Same as ***A***, but for a GAD65-positive neuron (wt mouse). ***D***, Summary data for CNQX-evoked currents. Box-and-whisker plots indicate the median (line), 25th and 75th percentile (box), range of data within 1.5× interquartile range (whiskers), and mean (white circles). Superimposed filled circles represent individual cells. A one-way ANOVA indicated a significant difference among the three groups (*F*_(2,32)_ = 16.97, *p* < 0.0001). ***p < 0.001 (pairwise differences). ***E***, Summary data for AMPA-evoked currents. Box-and-whisker plots as in ***E***.

Given the suggestion that γ-2 plays a role in hyperalgesia (Tao et al., 2006), we sought to determine whether Type I TARPs in SDH neurons are associated with functional AMPARs. We examined neurons in the outer region of lamina II, as these smaller excitatory cells were more likely to permit high-resolution whole-cell voltage-clamp recordings. We used the classical AMPAR blocker CNQX to identify TARP-associated AMPARs, as it acts as a partial agonist at AMPARs associated with Type I TARPs (γ-2, γ-3, γ-4, and γ-8) (Menuz et al., 2007; Bats et al., 2012). Because CNQX-evoked currents are generally small compared with those evoked by glutamate (Menuz et al., 2007), we included the positive allosteric modulator cyclothiazide (100 μm) in our solutions. We made recordings in the presence of TTX, gabazine, strychnine, and d-AP5 to block voltage-gated Na^+^ channels, GABA_A_ receptors, glycine receptors and NMDA receptors, respectively. As expected, bath application of CNQX reversibly blocked mEPSCs (Fig. 2*A–C*) in lamina II neurons from wt mice. CNQX also produced clear inward currents (29.5 ± 4.2 pA, *n* = 24, *p* < 0.0001 vs zero) that returned to baseline on washout (Fig. 2*A*). CNQX currents were also detectable in neurons from *stg/stg* mice lacking γ-2, but these were of much smaller amplitude than those in wt (4.0 ± 1.4 pA, *n* = 16, *p* = 0.014 vs zero, *p* < 0.0001 vs wt) (Fig. 2*B*,*D*). This striking attenuation suggests that γ-2 is normally the predominant Type I TARP associated with AMPARs in lamina II interneurons.

Approximately 20%–30% of lamina II neurons are inhibitory (Polgár et al., 2003; Santos et al., 2007; Yasaka et al., 2010), the majority being GABAergic (Zeilhofer et al., 2012). We next asked whether γ-2 is associated with AMPARs in these GABAergic interneurons. To address this, we measured CNQX-induced currents in cells identified in mice expressing GFP under the glutamic acid decarboxylase (GAD65) promoter (López-Bendito et al., 2004). In all GFP-positive cells where firing was examined (by depolarizing current injection; *n* = 10, data not shown), we observed a tonic firing pattern characteristic of GABAergic interneurons (Santos et al., 2007; Punnakkal et al., 2014). In GFP-positive cells, CNQX evoked a mean current of 5.1 ± 1.5 pA (*n* = 12, *p* = 0.0060 vs zero), markedly less than that in lamina II neurons of wt mice (*p* < 0.0001; Fig. 2*C*,*D*), raising the possibility of cell-type differences in TARP expression within lamina II.

TARP γ-2 plays an important role in many central neurons by regulating both AMPAR properties and their trafficking (Jackson and Nicoll, 2011b). The reduced magnitude of CNQX-evoked currents in *stg/stg* lamina II neurons indicates the presence of fewer Type I TARP-associated AMPARs at the cell surface. However, if cell surface expression of AMPARs in these neurons requires γ-2, then one would also expect a reduced response to AMPA itself. When we compared whole-cell current responses with bath-applied AMPA (20 μm) in lamina II neurons from *stg/stg* and wt mice, we found them to be of similar amplitude (1.5 ± 0.2 vs 1.6 ± 0.2 nA, *n* = 5 and *n* = 7 *stg/stg* and wt, respectively; *p* = 0.93) (Fig. 2*E*). This implies that γ-2, although normally associated with AMPARs in these cells, is not essential for their surface expression. In the absence of γ-2, surface receptors may be TARPless (Bats et al., 2012) or associated with other auxiliary subunits able to compensate for its loss (Menuz et al., 2008).

### Quantal EPSCs are attenuated in *stg*/*stg* lamina II neurons

**Figure 3.**
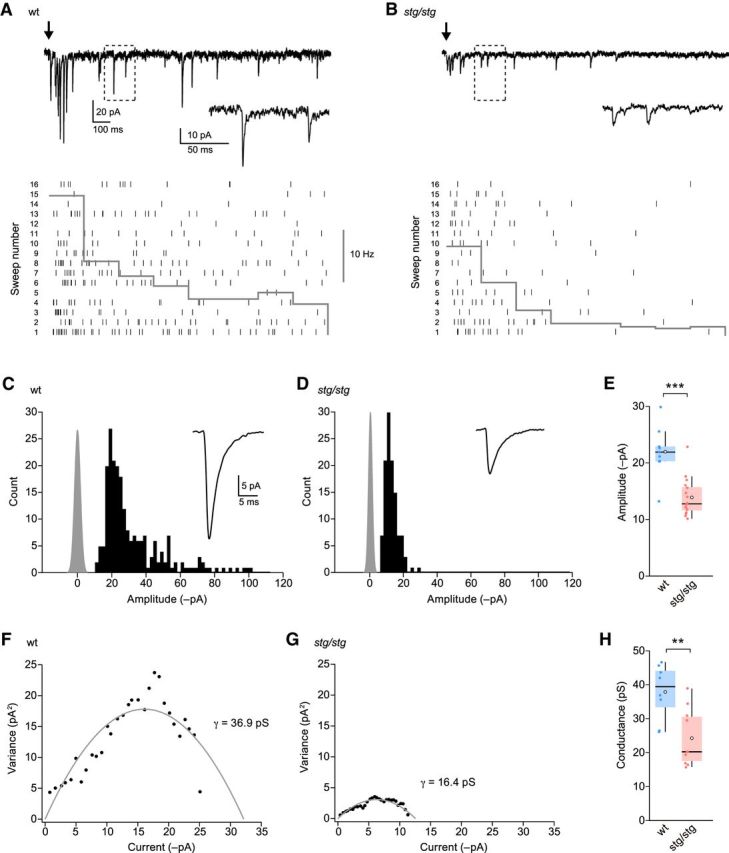
Evoked qEPSCs in lamina II neurons from wt and *stg/stg* mice. ***A***, Representative recording from a wt mouse of electrically evoked qEPSCs in the presence of SrCl_2_ (−60 mV). Black arrow indicates the time of stimulation. Inset, Expansion of indicated area. Bottom, Raster plot showing individual qEPSCs (vertical ticks) over 16 stimulation trials. Overlaid histogram shows the frequency of events within 200 ms bins averaged over the 16 trials. ***B***, Same as ***A***, but for a neuron from an *stg*/*stg* mouse. ***C***, Histogram of qEPSC amplitudes (same cell as ***A***). Gray represents background noise. Inset, Average mEPSC waveform. ***D***, Same as ***C***, but for the *stg/stg* neuron in ***B***. ***E***, Summary data for qEPSC amplitudes. ***F***, Current-variance relationship for the same cell as ***A***. The weighted mean conductance (γ) was determined from the parabolic fit (gray) of the data. ***G***, Same as ***F***, but for the *stg/*stg neuron in ***B***. ***H***, Summary data for estimated single-channel conductance. Box-and-whisker plots as in Figure 2. **p < 0.01. ***p < 0.001.

To determine whether γ-2 regulates synaptic AMPARs in lamina II neurons, we next compared the synaptic currents in cells from wt and *stg/stg* mice. To exclude possible presynaptic effects, we examined qEPSCs evoked in the presence of strontium (Goda and Stevens, 1994). In these conditions, local electrical stimulation promoted an asynchronous burst of qEPSCs in both wt and *stg/stg* (Fig. 3*A*,*B*). In cells from *stg/stg* mice, the average qEPSC amplitude was approximately halved (from 22.0 ± 1.5 to 12.5 ± 0.7 pA; *n* = 9 and 16, *p* = 0.00076) (Fig. 3*C–E*). This reduction in qEPSC amplitude could reflect a decrease in the number, conductance, or open probability of synaptic AMPARs. Using peak scaled psNSFA (Traynelis et al., 1993), we found that the weighted mean single-channel conductance of synaptic AMPARs was reduced in *stg/stg* cells compared with wt (from 37.9 ± 2.9 to 24.3 ± 2.7 pS; *n* = 8 and *n* = 10; *p* = 0.0097) (Fig. 3*F–H*). There was no difference in the estimated number of receptors open at the qEPSC peak (9.4 ± 1.1 vs 9.2 ± 0.8; *p* = 1.0). Thus, the reduced qEPSC amplitude in *stg/stg* appears to reflect reduced mean channel conductance rather than impaired AMPAR trafficking.

The frequency of evoked qEPSCs was reduced in *stg/stg* cells compared with wt (from 6.6 ± 1.0 to 3.3 ± 0.4 Hz, *n* = 9 and *n* = 16, respectively; *p* = 0.038). Although this difference could reflect an increase in the proportion of smaller amplitude events falling below the detection threshold, we cannot exclude a change in the number of synaptic inputs or our ability to stimulate these inputs (which may additionally vary depending on the size and position of the stimulating electrode). Interestingly, in *stg/stg* cells, we found no change in the qEPSC 20%–80% rise time (wt 0.38 ± 0.04 ms, *stg/stg* 0.41 ± 0.05 ms; *p* = 1.0) or τ_decay_ (3.4 ± 0.3 vs 3.0 ± 0.2 ms; *p* = 0.68). This is reminiscent of the situation previously described for qEPSCs and mEPSCs in cerebellar stellate cells from *stg*/*stg* mice, which are also reduced in amplitude but show only a modest (Jackson and Nicoll, 2011a) or no (Bats et al., 2012) change in decay kinetics. Although it is unclear whether the synaptic AMPARs remaining in lamina II neurons from *stg/stg* mice are associated with a TARP other than γ-2 or are TARPless, our results indicate that γ-2 is normally associated with AMPARs at synapses receiving locally evoked excitatory input.

### TARP γ-2 involvement in synaptic transmission is input-specific

**Figure 4.**
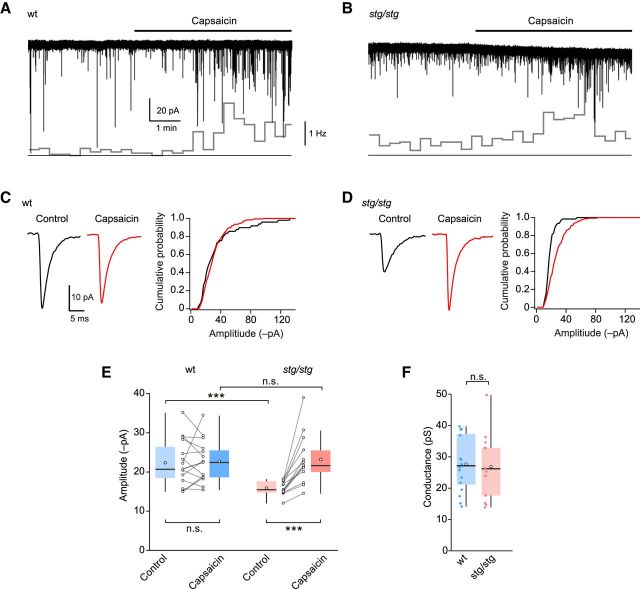
Basal, but not capsaicin-induced, mEPSCs are reduced in lamina II neurons from *stg/stg* mice. ***A***, Representative recording of mEPSCs from a lamina II neuron of a wt mouse showing the effect of bath-applied capsaicin (0.1 μm). Inset (bottom), Changes in mEPSC frequency (20 s bins). ***B***, Same as ***A***, but for a neuron from an *stg*/*stg* mouse. ***C***, Average mEPSC waveforms from the recording in ***A***, together with cumulative probability plots for the amplitudes of control and capsaicin-induced mEPSCs. ***D***, Same as ***C***, but for the neuron in ***B***. ***E***, Summary data for mEPSC amplitudes. A two-way “split plot” ANOVA indicated no significant main effect of genotype (*F*_(1,26)_ = 2.96, *p* = 0.098), a significant main effect of capsaicin treatment (*F*_(1,26)_ = 14.73, *p* = 0.00071), and a significant interaction between genotype and capsaicin treatment (*F*_(1,26)_ = 13.88, *p* = 0.00095). ***F***, Summary data for capsaicin-induced mEPSCs showing the weighted mean single-channel conductance from psNSFA. Box-and-whisker plots as in Figure 2. ***p < 0.001.

We next considered whether γ-2 is associated with AMPARs at synapses in those lamina II neurons that receive input from peripheral pain fibers. In individual cells from wt and *stg/stg* mice we first recorded mEPSCs under “basal” (control) conditions, where most events are thought to arise from non–C-fiber inputs (Yang and Li, 2001), and then in the presence of capsaicin to increase selectively the frequency of events arising from TRPV1-expressing C-fiber terminals (Yang et al., 1998). In slices from both wt and *stg/stg* mice, capsaicin (0.1 μm) increased the frequency of mEPSCs in approximately half the neurons tested (wt 17 of 28; *stg/stg* 13 of 33) (Fig. 4*A*,*B*). In the “capsaicin-sensitive” cells, mEPSC frequency was increased from 0.64 ± 0.13 Hz to 4.22 ± 1.35 Hz in wt (*p* = 0.024) and from 0.61 ± 0.23 to 2.57 ± 0.80 Hz in *stg/stg* (*p* = 0.017). Considering only capsaicin-sensitive cells, the average amplitude of control (“basal”) mEPSCs was less in *stg*/*stg* than in wt (15.9 ± 1.8 vs 22.4 ± 1.5 pA, *n* = 13 and *n* = 15; *p* = 0.00081) (Fig. 4*C–E*), consistent with the view that γ-2 normally plays a role at the putative non–C-fiber synapses from which “basal” mEPSCs are thought to originate. By contrast, the amplitudes of capsaicin-induced mEPSCs were similar in *stg/stg* and wt (23.2 ± 1.8 vs 22.7 ± 1.4 pA; *p* = 0.74) (Fig. 4*C–E*). Furthermore, psNSFA of the capsaicin-induced mEPSCs indicated no difference in mean channel conductance of synaptic AMPARs in *stg/stg* and wt cells (26.9 ± 2.9 vs 27.7 ± 2.2 pS, *n* = 13 and *n* = 16; *p* = 0.83; Fig. 4*F*). Together these observations suggest either that γ-2 is not normally associated with AMPARs at TRPV1-expressing C-fiber synapses or that other auxiliary subunits are present and can substitute for γ-2. In those cells where capsaicin failed to alter mEPSC frequency, the amplitude of the “basal” mEPSCs was similar in *stg/stg* and wt (18.3 ± 1.7 vs 17.5 ± 1.0 pA, *n* = 20 and *n* = 11; *p* = 1.0) and there was no difference in their frequency (0.95 ± 0.38 vs 0.55 ± 0.08 Hz; *p* = 0.86).

### γ-2 is preferentially expressed at non–C-fiber synapses

**Figure 5.**
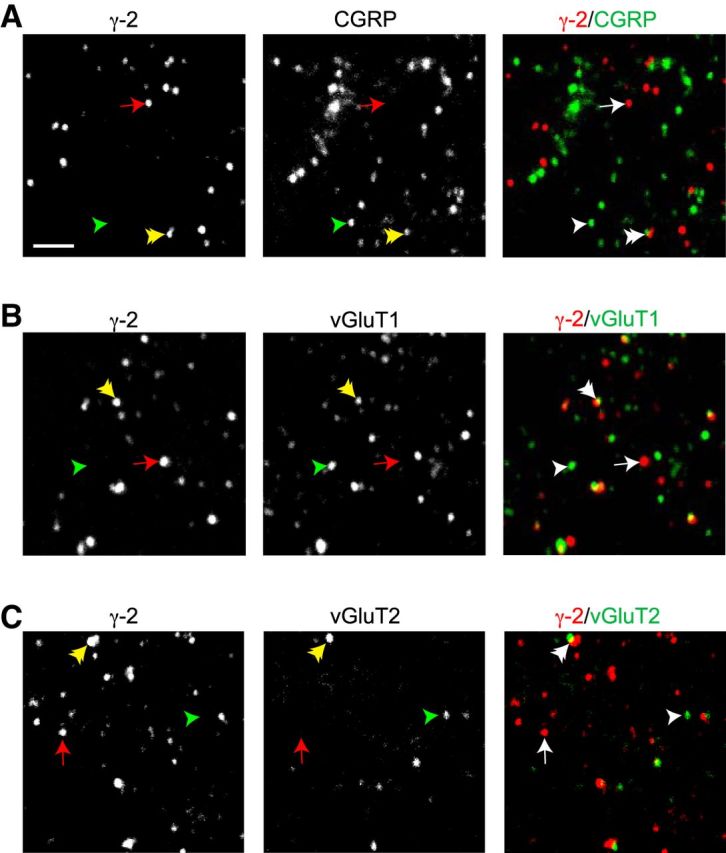
Synaptic expression pattern of γ-2 in outer lamina II. ***A***, Confocal images of immunolabeled γ-2 (left), CGRP (middle), and overlay (right). Red represents γ-2. Green represents CGRP. Overlay, Arrow indicates an example punctum positive for γ-2. Single arrowhead indicates example punctum positive for the presynaptic marker CGRP. Double arrowhead indicates an example of adjacent or overlapping γ-2 and CGRP puncta. In the individual images, the corresponding arrows and arrowheads are colored: red represents γ-2; green represents CGRP; yellow represents overlap. ***B***, Same as ***A***, but for the presynaptic protein vGluT1. ***C***, Same as ***A***, but for the presynaptic protein vGluT2. Images shown represent ∼3% of the area used for quantification of puncta. Scale bar, 2 μm.

To examine further our proposition that postsynaptic elements containing γ-2 were associated with non–C-fiber inputs, we imaged synaptic puncta in lamina II of spinal cord slices from 6 wt mice colabeled with antibodies against γ-2 and presynaptic marker proteins. We examined ROIs of 56.2 × 56.2 μm (∼3000 μm^2^) in outer lamina II and used an object detection algorithm (see Materials and Methods) to determine the proportion of γ-2 puncta that partially overlapped or were juxtaposed to presynaptic markers. In slices colabeled for γ-2 and the peptidergic C- and Aδ-fiber marker calcitonin gene-related peptide (CGRP) (Nagy et al., 2004), we identified a total of 3246 γ-2-positive puncta and 2370 CGRP-positive puncta (11 ROIs summed from six mice). The mean diameter of γ-2 and CGRP puncta was 390 and 448 nm, respectively. Adjusting for the chance probability of object overlap (see Materials and Methods), only 4.3 ± 0.9% of γ-2 puncta contacted CGRP-containing puncta. This indicates that only a small fraction of γ-2 puncta are contacted by peptidergic C- and Aδ-fibers. The corresponding analysis showed that 4.8 ± 1.1% of CGRP terminals contacted γ-2 puncta (Fig. 5*A*).

We also colabeled separate slices for γ-2 and the vesicular glutamate transporters vGluT1 and vGluT2, which are thought to belong to mutually exclusive classes of non–C-fiber terminals (Todd et al., 2003, but see Li et al., 2003) (Fig. 5*B*,*C*). As previously reported (Li et al., 2003), the expression of vGluT1 in outer lamina II was lower than that observed in deeper laminae. Nevertheless, at high magnification, we observed abundant vGluT1 puncta in this region. We identified a total of 1416 vGluT1-positive puncta (four ROIs from 4 mice) with a mean diameter of 503 nm and 2213 vGluT2-positive puncta (seven ROIs from 5 mice) with a mean diameter of 439 nm. Adjusting for the chance probability of object overlap, 38.5 ± 9.7% of γ-2 puncta were judged as contacting vGluT1-expressing terminals and 7.8 ± 1.6% were judged as contacting vGluT2-expressing terminals. Kruskal-Wallis one-way ANOVA indicated a significant difference in the probability of γ-2 overlap among the CGRP, vGluT1, and vGluT2 groups (*H*_2_ = 10.47, *p* = 0.0053) with pairwise Wilcoxon rank sum tests indicating *p* = 0.028 for CGRP versus vGluT1 and *p* = 0.055 for CGRP versus vGluT2. Together, these data suggest that γ-2 is preferentially adjacent to presumed non–CGRP-expressing, vGluT1-positive terminals, in keeping with our proposition that γ-2 is localized primarily at non–C-fiber synapses in lamina II.

### Peripheral inflammation promotes a switch from CI- to CP-AMPARs at C-fiber synapses

**Figure 6.**
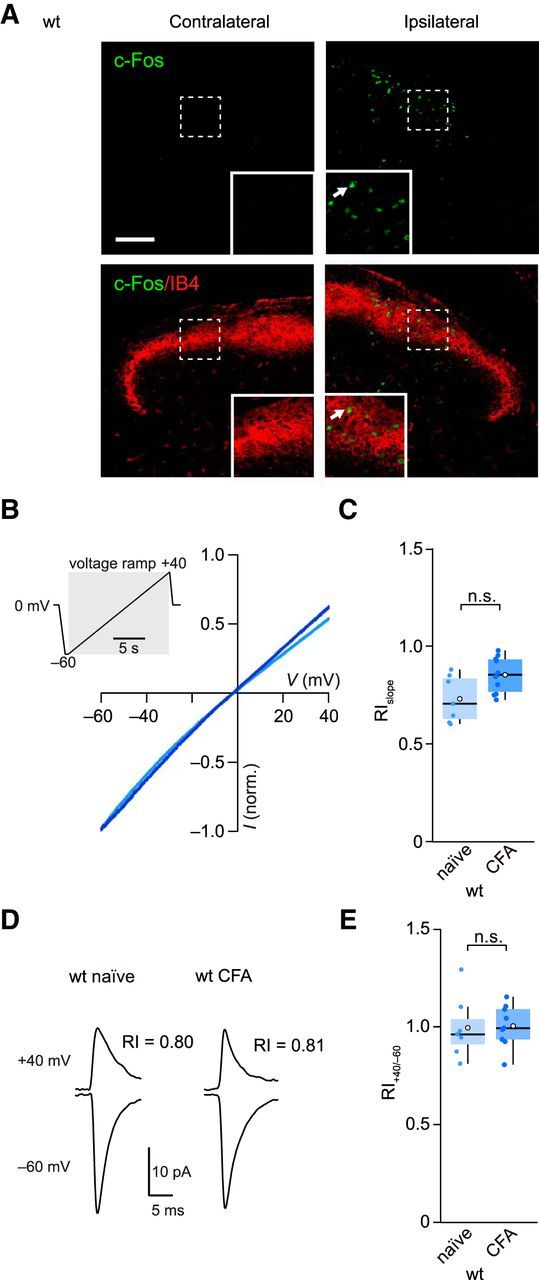
CFA treatment fails to alter AMPAR rectification in lamina II neurons. ***A***, Top, Representative immunolabeling (maximum intensity projection confocal images) showing CFA-induced c-Fos expression (green) in ipsilateral SDH 1.5 h following hindpaw injection in a wt mouse. Scale bar, 100 μm. Insets, Enlarged view from indicated area. White arrow indicates an example of a c-Fos-expressing cell. Bottom, Same as top, but with overlaid labeling for the lamina II marker IB4 (red). ***B***, Representative current-voltage relationships from lamina II neurons from a naive (light blue) and a CFA-treated mouse (dark blue) obtained 24 h after treatment. Currents were evoked by AMPA (20 μm) in the presence of cyclothiazide (100 μm). Inset, Voltage ramp protocol (−60 to 40 mV). ***C***, Summary data for RI_slope_ values (40/−60 mV). ***D***, Representative average of electrically evoked qEPSCs in SrCl_2_ recorded from lamina II neurons of a naive mouse and a CFA-treated mouse. RI_40/−60_ values indicated. ***E***, Summary RI_40/−60_ data for qEPSCs. Box-and-whisker plots as in Figure 2.

There is compelling evidence that exposure to noxious stimuli triggers upregulation of CP-AMPARs in SDH neurons that is thought to play a crucial role in hyperalgesia (Park et al., 2009; Kopach et al., 2011; Chen et al., 2013). However, it is unclear which synaptic inputs are affected and which TARPs are involved in these changes. To investigate this, we used a common model of chronic inflammatory pain by performing intraplantar injections of CFA (Pearson, 1956). We were able to confirm the effectiveness of our CFA injection by detecting the presence of a selectively ipsilateral increase in the expression of the immediate early gene c-Fos in the lumbar region (L3-L6) of SDH (Hossaini et al., 2014) (Fig. 6*A*).

**Figure 7.**
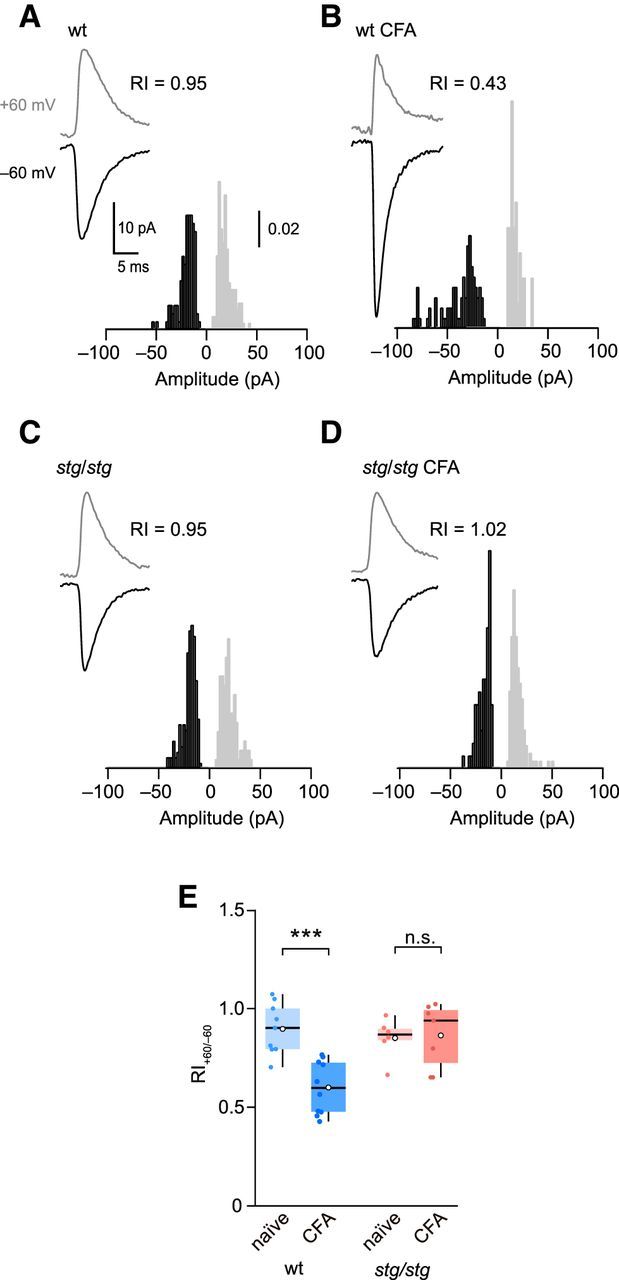
CFA treatment increases rectification of capsaicin-induced mEPSCs in lamina II neurons from wt but not *stg/stg* mice. ***A***, Representative averaged capsaicin-induced mEPSCs from a naive wt neuron; RI value indicated. Inset, Normalized amplitude histograms for the two voltages (60 mV gray, −60 mV black). ***B***, Same as ***A***, but for a lamina II neuron from a CFA-treated wt mouse. ***C***, Same as ***A***, but for a neuron from a naive *stg*/*stg* mouse. ***D***, Same as ***A***, but for a neuron from a CFA-treated *stg*/*stg* mouse. ***E***, Summary of RI_60/−60_ data for capsaicin-evoked mEPSCs. Box-and-whisker plots as in Figure 2. Two-way ANOVA showed no effect of genotype (*F*_(1,28)_ = 1.59, *p* = 0.22), a significant effect of CFA treatment (*F*_(1,28)_ = 11.39, *p* = 0.0022), and a significant interaction between genotype and treatment (*F*_(1,28)_ = 9.44, *p* = 0.0047). ***p < 0.001.

To test lamina II neurons for CFA-induced plasticity, we recorded from the corresponding ipsilateral dorsal horn and first examined whole-cell currents generated by bath applied AMPA (Fig. 6*B*). We made use of the fact that, when spermine is included in the patch pipette, CP-AMPARs display a characteristic inwardly rectifying current-voltage relationship that allows them to be readily identified (Bowie and Mayer, 1995; Kamboj et al., 1995; Koh et al., 1995). Previous studies had suggested that CFA treatment triggers an increase in rectification of AMPA-evoked currents in a tonically firing subset of lamina II neurons (Kopach et al., 2011). However, in our recordings with bath-applied AMPA, we found no evidence of an increase in rectification in cells from CFA-treated mice (RI_slope_ naive 0.73 ± 0.05, CFA 0.85 ± 0.03, *n* = 7 and *n* = 10; *p* = 0.13) (Fig. 6*C*). Similarly, the rectification of locally evoked qEPSCs remained unchanged (RI_40/−60_ 1.02 ± 0.05 vs 1.00 ± 0.04, *n* = 7 and *n* = 9; *p* = 0.96) (Fig. 6*D*,*E*). We next examined the capsaicin-induced mEPSCs thought to arise from C-fiber inputs. Following peripheral inflammation, these exhibited a marked increase in rectification (RI_60/−60_ decreased from 0.90 ± 0.04 to 0.59 ± 0.04, *n* = 9 naive and *n* = 10 CFA; *p* = 0.00016) (Fig. 7*A*,*B*,*E*). By contrast, in capsaicin-insensitive cells, the CFA treatment failed to alter rectification (RI_60/−60_ 0.93 ± 0.05 vs 0.86 ± 0.04, *n* = 5 naive and *n* = 8 CFA; *p* = 0.64). Together, these data suggest that the increased prevalence of CP-AMPARs that accompanies peripheral inflammation occurs specifically at pain fiber synapses.

### Upregulation of CP-AMPARs at C-fiber synapses requires γ-2

Behavioral studies have shown that γ-2 knockdown before the application of peripheral noxious stimuli lessens the severity of hyperalgesia (Tao et al., 2006; Guo et al., 2014). To test whether the upregulation of CP-AMPARs that we observed at C-fiber synapses following CFA treatment was dependent on γ-2, we repeated these experiments using *stg/stg* mice. As in wt animals, CFA injection increased c-Fos expression in the ipsilateral SDH (*n* = 3 mice; data not shown). However, in these *stg/stg* mice, CFA caused no change in the rectification of capsaicin-induced mEPSCs (RI_60/−60_, 0.85 ± 0.06 vs 0.88 ± 0.05, *n* = 6 naive and 7 CFA; *p* = 0.75) (Fig. 7*C–E*). This suggests that γ-2 is necessary for the CFA-induced upregulation of CP-AMPARs at these synapses in lamina II neurons.

Despite the necessity for γ-2 in our CFA-induced plasticity, in the naive animals, we found no difference between wt and *stg/stg* mice in the rectification of their capsaicin-induced mEPSCs (*p* = 0.47) (Fig. 7*E*). Neither was the rectification of whole-cell AMPA-evoked currents or that of evoked qEPSCs changed (*p* = 0.68 and *p* = 0.96, respectively; data not shown). Together, these results suggest that the absence of γ-2 does not enhance the prevalence of CP-AMPARs in lamina II neurons of naive mice. This is in striking contrast with cerebellar stellate cells, where increased AMPAR rectification is seen in *stg/stg* mice (Jackson and Nicoll, 2011a; Bats et al., 2012), and suggests that cell type-dependent differences in the AMPAR proteome influence the outcome of γ-2 deletion.

## Discussion

Our studies provide functional evidence for the involvement of an AMPAR auxiliary subunit in shaping excitatory transmission in spinal cord pain pathways. We establish that γ-2 contributes to the excitatory synaptic currents of lamina II neurons in an input-specific manner and is critical in CP-AMPAR plasticity at C-fiber synapses following peripheral inflammation.

### TARP expression in lamina II neurons

Our immunolabeling data showed γ-2 to be present in SDH lamina II of mouse, as previously observed in rat (Tao et al., 2006; Guo et al., 2014). We found that CNQX, a partial agonist at AMPARs associated with Type I TARPs (Menuz et al., 2007), induced a current in most lamina II neurons, and this was substantially reduced in neurons from *stg/stg* mice. Additionally, evoked qEPSCs were attenuated in *stg/stg* mice. These observations provide clear evidence for the presence of γ-2-associated AMPARs at synapses in these neurons. The fact that currents evoked by bath applied AMPA were not reduced in *stg/stg* mice might therefore appear unexpected. However, it is unlikely that activation of extrasynaptic and synaptic receptors would reveal differences if only a small subset of AMPARs (such as those at specific synaptic inputs) were impaired.

Although the CNQX-induced current in lamina II neurons was much smaller in *stg/stg* mice than in wt, the presence of a small residual current suggests that at least some AMPARs in these neurons may associate with Type I TARPs other than γ-2. These are most likely γ-4 or γ-8, as we detected protein for these in spinal cord samples. Nonspecific labeling prevented us from examining the distribution of γ-4 histologically, but its presence in lamina II of the rat (albeit without clear colocalization with AMPARs) has previously been reported (Larsson et al., 2013). We detected punctate immunoreactivity for γ-8 in lamina I and relatively diffuse labeling in lamina II, as also previously seen in rat (Larsson et al., 2013). Although it seems likely that γ-8 associates with functional AMPARs in lamina II neurons, studies in γ-8 knock-out mice (Rouach et al., 2005) would be necessary to address its significance in SDH pain circuitry.

Do Type II TARPs (γ-5 and γ-7) play any role in lamina II neurons? This is of interest as there is previous evidence for an association of these TARPs with CP-AMPARs (Soto et al., 2009; Bats et al., 2013). As CNQX does not act as an agonist at γ-7-associated AMPARs (Bats et al., 2012), it cannot be used to provide evidence for their presence. However, our failure to detect immunolabeling for γ-5 or γ-7 would argue against a role for either. Interestingly, γ-7 has been detected in lamina II of the rat, albeit not colocalized with AMPARs (Larsson et al., 2013). Whether this reflects a genuine species difference in TARP expression remains to be determined.

Although many of the lamina II neurons we examined are likely to have been excitatory (Todd and Sullivan, 1990; Polgár et al., 2003; Santos et al., 2007), we found that inhibitory interneurons within this lamina differed in their CNQX responses. Studies in other regions of the CNS have shown that GABAergic neurons can exhibit robust currents in response to CNQX (Menuz et al., 2007; Bats et al., 2012). In spinal cords from wt mice, GAD65-positive GABAergic neurons displayed, on average, smaller CNQX-induced current than the mixed population of neurons (expected to be predominantly excitatory). Although we cannot exclude a simple difference in AMPAR number, this observation raises the possibility of differential TARP expression. If this were the case, TARPs could influence the balance of excitation and inhibition within the SDH neuronal network (Braz et al., 2014), and their identification may aid in differentiating between SDH neuron types, an ongoing challenge in the field (Graham et al., 2007).

### Input-dependent TARP expression

In lamina II neurons from *stg/stg* mice, the amplitudes of qEPSCs evoked by local stimulation were reduced. Likewise, in a subset of lamina II cells receiving input from capsaicin-sensitive C-fibers, the amplitudes of basal mEPSCs, thought to arise from non–C-fiber inputs (Yang and Li, 2001), were reduced. However, capsaicin-induced mEPSCs (from C-fibers) within these same cells were unaltered in *stg/stg* mice. The most parsimonious explanation for these findings is that both qEPSCs and basal mEPSCs arise from the same inputs, which activate receptors associated with γ-2, whereas the synapses formed by TRPV1-expressing C-fibers on the same cell typically lack γ-2. In keeping with this view, we also found that γ-2-containing synapses in lamina II were rarely contacted by C-fibers (identified with CGRP or IB4) but exhibited a clear association with vGluT1-expressing non–C-fiber terminals. Thus, our functional and immunolabeling data are both consistent with the notion that γ-2 is expressed preferentially at non–C-fiber synapses.

### Input-specific CP-AMPAR plasticity

There is compelling evidence that AMPAR-mediated excitatory responses are augmented, and the CP-AMPAR contribution increased, in central sensitization (Latremoliere and Woolf, 2009). Moreover, blocking CP-AMPARs can limit the development of allodynia following peripheral injury (Sorkin et al., 1999) and can reverse hyperalgesia following peripheral inflammation (Sorkin et al., 2001). However, despite an established role for CP-AMPARs in pain hypersensitivity, surprisingly little is known about which cell types and synapses are modified. We found that CFA treatment increased the prevalence of CP-AMPARs at synapses formed by capsaicin-sensitive, TRPV1-expressing, C-fibers. Such a change was absent from other inputs on the cell that could be activated by focal stimulation, implying that the CFA-induced plasticity was synapse specific.

The extent to which homosynaptic, rather than heterosynaptic changes, contribute to central sensitization at activated pain fibers is unclear (Latremoliere and Woolf, 2009). Repeated stimulation of pain fibers is known to potentiate their postsynaptic responses (Ikeda et al., 2006), a prerequisite for homosynaptic plasticity. In line with such homosynaptic plasticity, intraplantar injection of capsaicin increases the relative abundance of GluA1 at a specific subset of C-fiber synapses in rat SDH neurons, favoring nonpeptidergic synapses over peptidergic ones (Larsson and Broman, 2008). However, it is of note that activity-dependent heterosynaptic changes have also been observed in SDH neurons (Thompson et al., 1993; Naka et al., 2013). Our finding that plasticity was present at synapses receiving input from TRPV1-expressing terminals, but not at other synapses on these cells, supports the view that homosynaptic plasticity plays a role in inflammatory pain.

### The importance of γ-2 for central sensitization

Knockdown of γ-2, before inflammation (Tao et al., 2006) or prior to plantar incision (Guo et al., 2014), has previously been shown to limit the severity of hyperalgesia. In the latter study, knockdown of γ-2 also precluded pain-induced increases in GluA1 surface expression. Our finding that peripheral inflammation no longer elicits an increase in CP-AMPARs at C-fiber synapses in *stg/stg* mice implies that γ-2 is required for the enhanced expression and localization of synaptic CP-AMPARs in this form of plasticity. In the brain, γ-2 is known to dynamically influence AMPAR trafficking (Tomita et al., 2005b; Bats et al., 2007; Opazo et al., 2010). Moreover, loss of γ-2 has been suggested to impair certain forms of synaptic plasticity involving CP-AMPARs (Jackson and Nicoll, 2011a). It therefore seems plausible that γ-2 within lamina II neurons plays an active role in mediating plasticity at C-fiber synapses, perhaps through changes in its interaction with PSD-95 and other postsynaptic proteins, as is observed with AMPARs following their pain-induced phosphorylation (Park et al., 2009).

The unusual arrangement, in which C-fiber AMPARs lack γ-2 in naive control mice but require γ-2 to undergo plasticity, suggests that induction of hyperalgesia involves γ-2-lacking AMPARs being replaced by γ-2-containing AMPARs. Interestingly, consistent with this hypothesis, hyperalgesia has been associated with increased coimmunoprecipitation of GluA1 with γ-2 in dorsal horn tissue extracts (Guo et al., 2014). It is noteworthy that the regulation of calcium-impermeable (CI−) and CP-AMPARs by TARPs appears to be cell-type dependent. For example, whereas γ-2 plays a role in the expression of CI-AMPARs in cerebellar stellate (Jackson and Nicoll, 2011a; Bats et al., 2012) and granule cells (Studniarczyk et al., 2013), in oligodendrocyte precursor cells (Zonouzi et al., 2011) it is required for the insertion of CP-AMPARs, similar to that observed in dorsal horn neurons. Clearly, it would now be of considerable interest to identify other auxiliary proteins that may account for the disparity between different cell types.

Our study establishes that TARP γ-2 serves a cell type- and input-specific role within lamina II of the dorsal horn. However, many questions remain about where precisely γ-2 and other auxiliary subunits are expressed within nociceptive pathways. Although genetic techniques have only begun to tease apart dorsal horn cell types and their connectivity, recent advances show promise (Peirs et al., 2015). It is noteworthy that an understanding of TARP expression in nociceptive circuitry may reveal novel therapeutic targets, in that TARPs endow AMPARs with a unique pharmacology. For example, two antagonists have recently been developed that selectively block AMPARs associated with γ-8 (Gardinier et al., 2016; Maher et al., 2016). Development of other TARP-selective drugs may increase the resolution of AMPAR-targeted pain therapies and aid in treating the maladaptive aspects of pain while retaining the basic nociceptive signaling necessary for survival.

## References

[B1] Bats C, Groc L, Choquet D (2007) The interaction between Stargazin and PSD-95 regulates AMPA receptor surface trafficking. Neuron 53:719–734. 10.1016/j.neuron.2007.01.030 17329211

[B2] Bats C, Soto D, Studniarczyk D, Farrant M, Cull-Candy SG (2012) Channel properties reveal differential expression of TARPed and TARPless AMPARs in stargazer neurons. Nat Neurosci 15:853–861. 10.1038/nn.3107 22581185 PMC3427011

[B3] Bats C, Farrant M, Cull-Candy SG (2013) A role of TARPs in the expression and plasticity of calcium-permeable AMPARs: evidence from cerebellar neurons and glia. Neuropharmacology 74:76–85. 10.1016/j.neuropharm.2013.03.037 23583927 PMC3751754

[B4] Bowie D, Mayer ML (1995) Inward rectification of both AMPA and kainate subtype glutamate receptors generated by polyamine-mediated ion channel block. Neuron 15:453–462. 10.1016/0896-6273(95)90049-7 7646897

[B5] Braz J, Solorzano C, Wang X, Basbaum AI (2014) Transmitting pain and itch messages: a contemporary view of the spinal cord circuits that generate gate control. Neuron 82:522–536. 10.1016/j.neuron.2014.01.018 24811377 PMC4492533

[B6] Carpenter AE, Jones TR, Lamprecht MR, Clarke C, Kang IH, Friman O, Guertin DA, Chang JH, Lindquist RA, Moffat J, Golland P, Sabatini DM (2006) CellProfiler: image analysis software for identifying and quantifying cell phenotypes. Genome Biol 7:R100. 10.1186/gb-2006-7-10-r100 17076895 PMC1794559

[B7] Chen SR, Zhou HY, Byun HS, Pan HL (2013) Nerve injury increases GluA2-lacking AMPA receptor prevalence in spinal cords: functional significance and signaling mechanisms. J Pharmacol Exp Ther 347:765–772. 10.1124/jpet.113.208363 24030012 PMC3836313

[B8] Coombs ID, Cull-Candy SG (2009) Transmembrane AMPA receptor regulatory proteins and AMPA receptor function in the cerebellum. Neuroscience 162:656–665. 10.1016/j.neuroscience.2009.01.004 19185052 PMC3217091

[B9] Engelman HS, Allen TB, MacDermott AB (1999) The distribution of neurons expressing calcium-permeable AMPA receptors in the superficial laminae of the spinal cord dorsal horn. J Neurosci 19:2081–2089. 10066261 10.1523/JNEUROSCI.19-06-02081.1999 PMC6782571

[B10] Fukaya M, Yamazaki M, Sakimura K, Watanabe M (2005) Spatial diversity in gene expression for VDCCgamma subunit family in developing and adult mouse brains. Neurosci Res 53:376–383. 10.1016/j.neures.2005.08.009 16171881

[B11] Gardinier KM, Gernert DL, Porter WJ, Reel JK, Ornstein PL, Spinazze P, Stevens FC, Hahn P, Hollinshead SP, Mayhugh D, Schkeryantz J, Khilevich A, De Frutos O, Gleason SD, Kato AS, Luffer-Atlas D, Desai PV, Swanson S, Burris KD, Ding C, et al (2016) Discovery of the first alpha-amino-3-hydroxy-5-methyl-4-isoxazolepropionic acid (AMPA) receptor antagonist dependent upon transmembrane AMPA receptor regulatory protein (TARP) γ-8. J Med Chem 59:4753–4768. 10.1021/acs.jmedchem.6b00125 27067148

[B12] Goda Y, Stevens CF (1994) Two components of transmitter release at a central synapse. Proc Natl Acad Sci U S A 91:12942–12946. 10.1073/pnas.91.26.12942 7809151 PMC45556

[B13] Graham BA, Brichta AM, Callister RJ (2007) Moving from an averaged to specific view of spinal cord pain processing circuits. J Neurophysiol 98:1057–1063. 10.1152/jn.00581.2007 17567772

[B14] Guo R, Zhao Y, Zhang M, Wang Y, Shi R, Liu Y, Xu J, Wu A, Yue Y, Wu J, Guan Y, Wang Y (2014) Down-regulation of Stargazin inhibits the enhanced surface delivery of alpha-amino-3-hydroxy-5-methyl-4-isoxazole propionate receptor GluR1 subunit in rat dorsal horn and ameliorates postoperative pain. Anesthesiology 121:609–619. 10.1097/ALN.0000000000000291 25093662 PMC4165695

[B15] Hartmann B, Ahmadi S, Heppenstall PA, Lewin GR, Schott C, Borchardt T, Seeburg PH, Zeilhofer HU, Sprengel R, Kuner R (2004) The AMPA receptor subunits GluR-A and GluR-B reciprocally modulate spinal synaptic plasticity and inflammatory pain. Neuron 44:637–650. 10.1016/j.neuron.2004.10.029 15541312

[B16] Hossaini M, Duraku LS, Kohli SK, Jongen JL, Holstege JC (2014) Spinal distribution of c-Fos activated neurons expressing enkephalin in acute and chronic pain models. Brain Res 1543:83–92. 10.1016/j.brainres.2013.10.044 24231552

[B17] Ikeda H, Stark J, Fischer H, Wagner M, Drdla R, Jäger T, Sandkühler J (2006) Synaptic amplifier of inflammatory pain in the spinal dorsal horn. Science 312:1659–1662. 10.1126/science.1127233 16778058

[B18] Jackson AC, Nicoll RA (2011a) Stargazin (TARP γ-2) is required for compartment-specific AMPA receptor trafficking and synaptic plasticity in cerebellar stellate cells. J Neurosci 31:3939–3952. 10.1523/JNEUROSCI.5134-10.2011 21411637 PMC3104604

[B19] Jackson AC, Nicoll RA (2011b) The expanding social network of ionotropic glutamate receptors: TARPs and other transmembrane auxiliary subunits. Neuron 70:178–199. 10.1016/j.neuron.2011.04.007 21521608 PMC3119519

[B20] Kamboj SK, Swanson GT, Cull-Candy SG (1995) Intracellular spermine confers rectification on rat calcium-permeable AMPA and kainate receptors. J Physiol 486:297–303. 10.1113/jphysiol.1995.sp020812 7473197 PMC1156521

[B21] Kamentsky L, Jones TR, Fraser A, Bray MA, Logan DJ, Madden KL, Ljosa V, Rueden C, Eliceiri KW, Carpenter AE (2011) Improved structure, function and compatibility for CellProfiler: modular high-throughput image analysis software. Bioinformatics 27:1179–1180. 10.1093/bioinformatics/btr095 21349861 PMC3072555

[B22] Katano T, Furue H, Okuda-Ashitaka E, Tagaya M, Watanabe M, Yoshimura M, Ito S (2008) N-ethylmaleimide-sensitive fusion protein (NSF) is involved in central sensitization in the spinal cord through GluR2 subunit composition switch after inflammation. Eur J Neurosci 27:3161–3170. 10.1111/j.1460-9568.2008.06293.x 18598260

[B23] Koh DS, Geiger JR, Jonas P, Sakmann B (1995) Ca^2+^-permeable AMPA and NMDA receptor channels in basket cells of rat hippocampal dentate gyrus. J Physiol 485:383–402. 10.1113/jphysiol.1995.sp020737 7545230 PMC1158000

[B24] Kopach O, Kao SC, Petralia RS, Belan P, Tao YX, Voitenko N (2011) Inflammation alters trafficking of extrasynaptic AMPA receptors in tonically firing lamina II neurons of the rat spinal dorsal horn. Pain 152:912–923. 10.1016/j.pain.2011.01.016 21282008 PMC3079375

[B25] Kudoh SN, Taguchi T (2002) A simple exploratory algorithm for the accurate and fast detection of spontaneous synaptic events. Biosens Bioelectron 17:773–782. 10.1016/S0956-5663(02)00053-2 12191925

[B26] Larsson M, Broman J (2008) Translocation of GluR1-containing AMPA receptors to a spinal nociceptive synapse during acute noxious stimulation. J Neurosci 28:7084–7090. 10.1523/JNEUROSCI.5749-07.2008 18614677 PMC6670484

[B27] Larsson M, Agalave N, Watanabe M, Svensson CI (2013) Distribution of transmembrane AMPA receptor regulatory protein (TARP) isoforms in the rat spinal cord. Neuroscience 248:180–193. 10.1016/j.neuroscience.2013.05.060 23751177

[B28] Latremoliere A, Woolf CJ (2009) Central sensitization: a generator of pain hypersensitivity by central neural plasticity. J Pain 10:895–926. 10.1016/j.jpain.2009.06.012 19712899 PMC2750819

[B29] Letts VA, Felix R, Biddlecome GH, Arikkath J, Mahaffey CL, Valenzuela A, Bartlett FS 2nd, Mori Y, Campbell KP, Frankel WN (1998) The mouse *stargazer* gene encodes a neuronal Ca^2+^-channel gamma subunit. Nat Genet 19:340–347. 10.1038/1228 9697694

[B30] Li JL, Fujiyama F, Kaneko T, Mizuno N (2003) Expression of vesicular glutamate transporters, VGluT1 and VGluT2, in axon terminals of nociceptive primary afferent fibers in the superficial layers of the medullary and spinal dorsal horns of the rat. J Comp Neurol 457:236–249. 10.1002/cne.10556 12541308

[B31] López-Bendito G, Sturgess K, Erdélyi F, Szabó G, Molnár Z, Paulsen O (2004) Preferential origin and layer destination of GAD65-GFP cortical interneurons. Cereb Cortex 14:1122–1133. 10.1093/cercor/bhh072 15115742

[B32] Maher MP, Wu N, Ravula S, Ameriks MK, Savall BM, Liu C, Lord B, Wyatt RM, Matta JA, Dugovic C, Yun S, Ver Donck L, Steckler T, Wickenden AD, Carruthers NI, Lovenberg TW (2016) Discovery and characterization of AMPA receptor modulators selective for TARP-γ8. J Pharmacol Exp Ther 357:394–414. 10.1124/jpet.115.231712 26989142

[B33] Menuz K, Stroud RM, Nicoll RA, Hays FA (2007) TARP auxiliary subunits switch AMPA receptor antagonists into partial agonists. Science 318:815–817. 10.1126/science.1146317 17975069

[B34] Menuz K, O'Brien JL, Karmizadegan S, Bredt DS, Nicoll RA (2008) TARP redundancy is critical for maintaining AMPA receptor function. J Neurosci 28:8740–8746. 10.1523/JNEUROSCI.1319-08.2008 18753375 PMC3159041

[B35] Moffett JR, Namboodiri MA, Neale JH (1993) Enhanced carbodiimide fixation for immunohistochemistry: application to the comparative distributions of N-acetylaspartylglutamate and N-acetylaspartate immunoreactivities in rat brain. J Histochem Cytochem 41:559–570. 10.1177/41.4.8450195 8450195

[B36] Nagy GG, Al-Ayyan M, Andrew D, Fukaya M, Watanabe M, Todd AJ (2004) Widespread expression of the AMPA receptor GluR2 subunit at glutamatergic synapses in the rat spinal cord and phosphorylation of GluR1 in response to noxious stimulation revealed with an antigen-unmasking method. J Neurosci 24:5766–5777. 10.1523/JNEUROSCI.1237-04.2004 15215299 PMC6729210

[B37] Nagy I, Woolf CJ, Dray A, Urbán L (1994) Cobalt accumulation in neurons expressing ionotropic excitatory amino acid receptors in young rat spinal cord: morphology and distribution. J Comp Neurol 344:321–335. 10.1002/cne.903440302 8063957

[B38] Naka A, Gruber-Schoffnegger D, Sandkühler J (2013) Non-Hebbian plasticity at C-fiber synapses in rat spinal cord lamina I neurons. Pain 154:1333–1342. 10.1016/j.pain.2013.04.011 23707311 PMC3708128

[B39] Nissenbaum J, Devor M, Seltzer Z, Gebauer M, Michaelis M, Tal M, Dorfman R, Abitbul-Yarkoni M, Lu Y, Elahipanah T, delCanho S, Minert A, Fried K, Persson AK, Shpigler H, Shabo E, Yakir B, Pisanté A, Darvasi A (2010) Susceptibility to chronic pain following nerve injury is genetically affected by CACNG2. Genome Res 20:1180–1190. 10.1101/gr.104976.110 20688780 PMC2928496

[B40] Opazo P, Labrecque S, Tigaret CM, Frouin A, Wiseman PW, De Koninck P, Choquet D (2010) CaMKII triggers the diffusional trapping of surface AMPARs through phosphorylation of stargazin. Neuron 67:239–252. 10.1016/j.neuron.2010.06.007 20670832

[B41] Park JS, Voitenko N, Petralia RS, Guan X, Xu JT, Steinberg JP, Takamiya K, Sotnik A, Kopach O, Huganir RL, Tao YX (2009) Persistent inflammation induces GluR2 internalization via NMDA receptor-triggered PKC activation in dorsal horn neurons. J Neurosci 29:3206–3219. 10.1523/JNEUROSCI.4514-08.2009 19279258 PMC2664544

[B42] Pearson CM (1956) Development of arthritis, periarthritis and periostitis in rats given adjuvants. Proc Soc Exp Biol Med 91:95–101. 10.3181/00379727-91-22179 13297719

[B43] Peirs C, Williams SP, Zhao X, Walsh CE, Gedeon JY, Cagle NE, Goldring AC, Hioki H, Liu Z, Marell PS, Seal RP (2015) Dorsal horn circuits for persistent mechanical pain. Neuron 87:797–812. 10.1016/j.neuron.2015.07.029 26291162 PMC4562334

[B44] Polgár E, Hughes DI, Riddell JS, Maxwell DJ, Puskár Z, Todd AJ (2003) Selective loss of spinal GABAergic or glycinergic neurons is not necessary for development of thermal hyperalgesia in the chronic constriction injury model of neuropathic pain. Pain 104:229–239. 10.1016/S0304-3959(03)00011-3 12855333

[B45] Polgár E, Watanabe M, Hartmann B, Grant SG, Todd AJ (2008) Expression of AMPA receptor subunits at synapses in laminae I-III of the rodent spinal dorsal horn. Mol Pain 4:5. 10.1186/1744-8069-4-5 18215271 PMC2248168

[B46] Punnakkal P, von Schoultz C, Haenraets K, Wildner H, Zeilhofer HU (2014) Morphological, biophysical and synaptic properties of glutamatergic neurons of the mouse spinal dorsal horn. J Physiol 592:759–776. 10.1113/jphysiol.2013.264937 24324003 PMC3934713

[B47] Rigby M, Cull-Candy SG, Farrant M (2015) Transmembrane AMPAR regulatory protein γ-2 is required for the modulation of GABA release by presynaptic AMPARs. J Neurosci 35:4203–4214. 10.1523/JNEUROSCI.4075-14.2015 25762667 PMC4355196

[B48] Rouach N, Byrd K, Petralia RS, Elias GM, Adesnik H, Tomita S, Karimzadegan S, Kealey C, Bredt DS, Nicoll RA (2005) TARP γ-8 controls hippocampal AMPA receptor number, distribution and synaptic plasticity. Nat Neurosci 8:1525–1533. 10.1038/nn1551 16222232

[B49] Santos SF, Rebelo S, Derkach VA, Safronov BV (2007) Excitatory interneurons dominate sensory processing in the spinal substantia gelatinosa of rat. J Physiol 581:241–254. 10.1113/jphysiol.2006.126912 17331995 PMC2075233

[B50] Silverman JD, Kruger L (1990) Selective neuronal glycoconjugate expression in sensory and autonomic ganglia: relation of lectin reactivity to peptide and enzyme markers. J Neurocytol 19:789–801. 10.1007/BF01188046 2077115

[B51] Sorkin LS, Yaksh TL, Doom CM (1999) Mechanical allodynia in rats is blocked by a Ca^2+^ permeable AMPA receptor antagonist. Neuroreport 10:3523–3526. 10.1097/00001756-199911260-00011 10619637

[B52] Sorkin LS, Yaksh TL, Doom CM (2001) Pain models display differential sensitivity to Ca^2+^-permeable non-NMDA glutamate receptor antagonists. Anesthesiology 95:965–973. 10.1097/00000542-200110000-00028 11605940

[B53] Soto D, Coombs ID, Renzi M, Zonouzi M, Farrant M, Cull-Candy SG (2009) Selective regulation of long-form calcium-permeable AMPA receptors by an atypical TARP, γ-5. Nat Neurosci 12:277–285. 10.1038/nn.2266 19234459 PMC2735763

[B54] Studniarczyk D, Coombs I, Cull-Candy SG, Farrant M (2013) TARP γ-7 selectively enhances synaptic expression of calcium-permeable AMPARs. Nat Neurosci 16:1266–1274. 10.1038/nn.3473 23872597 PMC3858651

[B55] Tao F, Skinner J, Su Q, Johns RA (2006) New role for spinal Stargazin in alpha-amino-3-hydroxy-5-methyl-4-isoxazolepropionic acid receptor-mediated pain sensitization after inflammation. J Neurosci Res 84:867–873. 10.1002/jnr.20973 16791853

[B56] Thompson SW, Woolf CJ, Sivilotti LG (1993) Small-caliber afferent inputs produce a heterosynaptic facilitation of the synaptic responses evoked by primary afferent A-fibers in the neonatal rat spinal cord in vitro. J Neurophysiol 69:2116–2128. 8350135 10.1152/jn.1993.69.6.2116

[B57] Todd AJ (2010) Neuronal circuitry for pain processing in the dorsal horn. Nat Rev Neurosci 11:823–836. 10.1038/nrn2947 21068766 PMC3277941

[B58] Todd AJ, Sullivan AC (1990) Light microscope study of the coexistence of GABA-like and glycine-like immunoreactivities in the spinal cord of the rat. J Comp Neurol 296:496–505. 10.1002/cne.902960312 2358549

[B59] Todd AJ, Hughes DI, Polgár E, Nagy GG, Mackie M, Ottersen OP, Maxwell DJ (2003) The expression of vesicular glutamate transporters VGLUT1 and VGLUT2 in neurochemically defined axonal populations in the rat spinal cord with emphasis on the dorsal horn. Eur J Neurosci 17:13–27. 10.1046/j.1460-9568.2003.02406.x 12534965

[B60] Tomita S, Chen L, Kawasaki Y, Petralia RS, Wenthold RJ, Nicoll RA, Bredt DS (2003) Functional studies and distribution define a family of transmembrane AMPA receptor regulatory proteins. J Cell Biol 161:805–816. 10.1083/jcb.200212116 12771129 PMC2199354

[B61] Tomita S, Stein V, Stocker TJ, Nicoll RA, Bredt DS (2005a) Bidirectional synaptic plasticity regulated by phosphorylation of stargazin-like TARPs. Neuron 45:269–277. 10.1016/j.neuron.2005.01.009 15664178

[B62] Tomita S, Adesnik H, Sekiguchi M, Zhang W, Wada K, Howe JR, Nicoll RA, Bredt DS (2005b) Stargazin modulates AMPA receptor gating and trafficking by distinct domains. Nature 435:1052–1058. 10.1038/nature03624 15858532

[B63] Tong CK, MacDermott AB (2006) Both Ca^2+^-permeable and -impermeable AMPA receptors contribute to primary synaptic drive onto rat dorsal horn neurons. J Physiol 575:133–144. 10.1113/jphysiol.2006.110072 16763002 PMC1819427

[B64] Traynelis SF, Silver RA, Cull-Candy SG (1993) Estimated conductance of glutamate receptor channels activated during EPSCs at the cerebellar mossy fiber-granule cell synapse. Neuron 11:279–289. 10.1016/0896-6273(93)90184-S 7688973

[B65] Traynelis SF, Wollmuth LP, McBain CJ, Menniti FS, Vance KM, Ogden KK, Hansen KB, Yuan H, Myers SJ, Dingledine R (2010) Glutamate receptor ion channels: structure, regulation, and function. Pharmacol Rev 62:405–496. 10.1124/pr.109.002451 20716669 PMC2964903

[B66] Vikman KS, Rycroft BK, Christie MJ (2008) Switch to Ca^2+^-permeable AMPA and reduced NR2B NMDA receptor-mediated neurotransmission at dorsal horn nociceptive synapses during inflammatory pain in the rat. J Physiol 586:515–527. 10.1113/jphysiol.2007.145581 18033811 PMC2375596

[B67] Wang X, Zhang J, Eberhart D, Urban R, Meda K, Solorzano C, Yamanaka H, Rice D, Basbaum AI (2013) Excitatory superficial dorsal horn interneurons are functionally heterogeneous and required for the full behavioral expression of pain and itch. Neuron 78:312–324. 10.1016/j.neuron.2013.03.001 23622066 PMC3700415

[B68] Wang Y, Mu X, Wu J, Wu A, Fang L, Li J, Yue Y (2011) Differential roles of phosphorylated AMPA receptor GluR1 subunits at Serine-831 and Serine-845 sites in spinal cord dorsal horn in a rat model of post-operative pain. Neurochem Res 36:170–176. 10.1007/s11064-010-0288-y 20953906

[B69] Watanabe M, Fukaya M, Sakimura K, Manabe T, Mishina M, Inoue Y (1998) Selective scarcity of NMDA receptor channel subunits in the stratum lucidum (mossy fibre-recipient layer) of the mouse hippocampal CA3 subfield. Eur J Neurosci 10:478–487. 10.1046/j.1460-9568.1998.00063.x 9749710

[B70] Yamazaki M, Fukaya M, Hashimoto K, Yamasaki M, Tsujita M, Itakura M, Abe M, Natsume R, Takahashi M, Kano M, Sakimura K, Watanabe M (2010) TARPs γ-2 and γ-7 are essential for AMPA receptor expression in the cerebellum. Eur J Neurosci 31:2204–2220. 10.1111/j.1460-9568.2010.07254.x 20529126

[B71] Yang K, Li YQ (2001) Origins of spontaneous and noxious stimuli-evoked miniature EPSCs in substantia gelatinosa. Neuroreport 12:39–42. 10.1097/00001756-200101220-00016 11201088

[B72] Yang K, Kumamoto E, Furue H, Yoshimura M (1998) Capsaicin facilitates excitatory but not inhibitory synaptic transmission in substantia gelatinosa of the rat spinal cord. Neurosci Lett 255:135–138. 10.1016/S0304-3940(98)00730-7 9832191

[B73] Yasaka T, Tiong SY, Hughes DI, Riddell JS, Todd AJ (2010) Populations of inhibitory and excitatory interneurons in lamina II of the adult rat spinal dorsal horn revealed by a combined electrophysiological and anatomical approach. Pain 151:475–488. 10.1016/j.pain.2010.08.008 20817353 PMC3170912

[B74] Yasaka T, Tiong SY, Polgár E, Watanabe M, Kumamoto E, Riddell JS, Todd AJ (2014) A putative relay circuit providing low-threshold mechanoreceptive input to lamina I projection neurons via vertical cells in lamina II of the rat dorsal horn. Mol Pain 10:3. 10.1186/1744-8069-10-3 24433581 PMC3897975

[B75] Zeilhofer HU, Wildner H, Yévenes GE (2012) Fast synaptic inhibition in spinal sensory processing and pain control. Physiol Rev 92:193–235. 10.1152/physrev.00043.2010 22298656 PMC3590010

[B76] Zonouzi M, Renzi M, Farrant M, Cull-Candy SG (2011) Bidirectional plasticity of calcium-permeable AMPA receptors in oligodendrocyte lineage cells. Nat Neurosci 14:1430–1438. 10.1038/nn.2942 21983683 PMC3204222

